# The Teratogenicity and the Action Mechanism of Gallic Acid Relating with Brain and Cervical Muscles

**DOI:** 10.1371/journal.pone.0119516

**Published:** 2015-06-01

**Authors:** Chiu Lan Hsieh, Chien-Hong Lin, Kuan Chou Chen, Chiung-Chi Peng, Robert Y. Peng

**Affiliations:** 1 Graduate Institute of Biotechnology, Changhua University of Education, Changhua, Taiwan; 2 Department of Urology, School of Medicine, College of Medicine, Taipei Medical University, Taipei, Taiwan; 3 Department of Urology, Shuang Ho Hospital, Taipei Medical University, New Taipei City, Taiwan; 4 Graduate Institute of Clinical Medicine, College of Medicine, Taipei Medical University, Taipei, Taiwan; 5 Research Institute of Biotechnology, Hungkuang University, Taichung City, Taiwan; Taipei Medical University, TAIWAN

## Abstract

Gallic acid (3,4,5-trihydroxybenzoic acid) (GA) and other flavanoids are extensively used in nutraceuticals because of their antioxidant and antiinflammatory properties. While examining whether GA is effective in alleviating valproic-acid-induced teratogenesis in a chicken embryo model (CEM), we observed embryo hemorrhage and liposis in the musculi longissimus cervicis. We conducted this study to determine whether GA is inherently teratogenic and the extent to which the risk can be transferred to fetuses. A CEM was used to administer GA at 2, 6, 10, and 14 μM. GA at 2 μM did not exhibit cytotoxicity. At 6, 10, and 14 μM, GA caused severe decreases in body and liver weights, causing -5.6%, -21.3%, and -27.5% body weights and 4.0, 3.8, and 3.2-g, liver weights, respectively, in day-1 chicks. The optimal alive birth rate (or damaging rate) reached 33.3%, 39.4%, and 29.2% at 6, 10, and 14 μM GA, respectively. The damaged tissue was primarily cervical muscle (musculi longissimus cervicis), as evidenced by liposis, Zenker’s necrosis, and hemolysis. The erythrocyte, hemoglobin, eosinophil, lymphocyte, and monocyte counts were severely reduced and PPAR-α was downregulated, whereas the Ras/Raf/JAK/STAT pathway was upregulated. The GA dose required to induce teratogenesis was ≥ 6 μM (1.02 mg/kg), which can be easily consumed by pregnant women in typical teas such as Chinese Pu-’Er and Chinese black teas, indicating a potential risk to human fetuses. GA at doses ≥ 1.02 mg/kg of body weight potentially causes characteristic cerebral hemolysis and liposis in the musculi longissimus cervicis. The mechanism of action of GA is multidisciplinary: The liposis can be ascribed to downregulation of PPAR-α; the erythrocyte hemolysis can be attributed to its unique autooxidative and prooxidant behavior and the inhibition of carbonic anhydrase; and the proliferation and differentiation deficits can be attributed to the upregulation of the Ras/Raf/JAK/STAT pathway.

## Introduction

Gallic acid (3,4,5-trihydroxybenzoic acid) (GA), a polyphenolic compound, is extensively used in nutraceuticals because of its antioxidant and antiinflammatory properties [1–5]. GA exhibits renal protection against chronic kidney disease (CKD) [6], and quercetin has raised warning to CKD if admitted to long-term administration [7]. Previously, we established the ovo chicken embryo model (CEM) to investigate the teratogenicity of valproic acid (VPA) [8]. When we used the CEM to evaluate the antiteratogenic bioactivity of polyphenolic nutraceuticals, we observed severe hemolysis and accumulated liposis in the musculi longissimus cervicis, which occurred in a dose-responsive manner. Because GA is widely used in numerous foods and drinks, such as oolong tea, higher risks can be raised by the dietary uptake, particularly in tea beverages. The GA content in various tea preparations (in gkg^-1^ on dry basis) are as follows: Chinese green teas (5.2 ± 0.3), Chinese Pu-’Er tea (14.9 ± 0.4), and black teas (3.2–3.6) [9]. These dietary hydroxybenzoic acid derivatives can elicit extra burdens on specific subjects, such as CKD patients and embryos [10].

Recently, Ko et al. demonstrated *in vitro* that glucose-6-phosphate dehydrogenase (G6PD)-deficient subjects are vulnerable to chemical-induced hemolysis if exposed to oxidative agents [11]. Thus far, most cited GA effects have been positive regarding antiteratogenesis [12, 13]; only a few studies have implicated negative effects caused by its prooxidative activity, including damage to aorta vascular smooth muscle cells (VSMCs) [14] and cytotoxic effects on testicular cells [15]. GA (but not quercetin) inhibits gap-junction intercellular communication (GJIC; a carcinogenic phenomenon), which is only partially reduced by catalase [16]. GA is ineffective at protecting PC12 cells from death caused by H_2_O_2_ attacks [17]. GA-induced cleavage of poly (ADP-ribose) polymerase (PARP) is strongly related to apoptosis in neurons [18]. GA downregulates Bcl-2 in PC12 cells through the combined effects of dietary phenolics and reactive oxygen species (ROS) on oxidative neuronal cell death [18]. Thus far, few studies have evaluated the teratogenic potential of gallic acid. To determine whether GA is potentially teratogenic and can induce hemorrhages, the CEM was adopted to conduct an *in vivo* study. Daily GA consumption was evaluated based on an assumed daily intake of 5 g of desiccated tea powder [9]. We examined the malformation rate (including the hemorrhage rate), pathological changes of the cervical muscle, inflammatory cytokines affected, including TNF-α and IL-6, and antioxidative markers, including ROS and MDA. Moreover, because the Ras/Raf/JAK/STAT pathway is related to skeletal muscle regeneration [19] and Lo et al. reported that GA kills melanoma cell lines through the Ras/JAK/STAT pathway [20], this signal pathway was also carefully analyzed using a western blot.

## Materials and Methods

### Ethics Statement

All experiments were conducted with respect for the principles of laboratory animal care and were consistent with the Taiwan Animal Protection Law (1998). This included official approval from the Animal Ethics Committee of Changhua University of Education, the level of expected mortality was considered.

### Chemicals

GA was obtained from Dainippon Pharmaceutical Co., Ltd. (Osaka, Japan) as a yellowish–white crystalline powder with a purity of over 98%. Normal saline was purchased fro China Pharmaceutical and Chemical Co. (Taipei, Taiwan).

Aβ_25–35_, 3-(4, 5-dimethylthiazol-2-yl)-2, 5-di-phenyl (MTT), Folin-Ciocalteau reagent (50%) was purchased from Sigma (St. Louis, MO, USA). Rabbit antibodies to cervical muscle tissue Ras (1:1000), Raf (1:1000), GRB2 (1:1000), LIFR (1:1000), gp130 (1:1000), STAT (1:1000), TNF-α (1:1000), peroxisome proliferator activated receptor alpha (PPAR-α) (1:1000), and β-actin, and the secondary antibodies were provided by Santa Cruz Biotechnology (Santa Cruz, CA, USA). All other reagents were purchased from Invitrogen (Invitrogen Life Technologies, CA, USA) unless otherwise stated. ELISA IL-6, ROS, and MDA kits were supplied by R&D Systems (Boston, MA, USA).

### Estimation of the appropriate experimental dosage of gallic acid

According to the data of Lin et al. [9], a daily consumption of 5 g of Chinese Pu-’Er tea contains 74.5 mg of GA (or 1.24 mg/kg), corresponding to a serum level of 18.6 mg/L (109.3 μM), based on an assumed total blood volume of 4 L for a pregnant woman. Similarly, 17 mg of GA (or 0.28 mg/kg) in Chinese black tea yields a serum level 4.25 mg/L or 25 μM. Thus, a fetus can absorb highly concentrated GA if it is not rapidly degraded by maternal metabolism. Following the study of Niho et al. [21], we proposed an experimental dose of 2 to 14 μM; any GA toxicity observed in the experiment would, therefore, be far less than that estimated for the daily consumption of 5 g of Chinese tea.

### Source of fertilized eggs and experimental protocol

One hundred and eighty day-1 fertilized Leghorn eggs, each weighing 50 ± 2.5 g, were provided by Qing-Dang Chicken Farm (Taichung, Taiwan). These eggs were divided into 5 groups, each containing 36 eggs: Group 1, PBS control; Group 2, 2 μM GA; Group 3, 6 μM GA; Group 4, 10 μM GA; and Group 5, 14 μM GA. Each group was further divided into 3 subgroups, each containing 12 fertilized eggs.

In the Hamilton-Hamburger (HH) stages [22], somite formation and neural folds occur early from stage 7 to stage 21, which corresponds to 23 h to 3.5 d. A blood island with 4 somites develops at Stage 8 (26–29 h). From day 9.5 to day 10, primitive chick morphology can be clearly observed, and, during this period, the length of the third toe from the tip to the middle of the metatarsal joint can reach 5.4 ± 0.3 mm; the length of the beak from the anterior angle of the nostril to the tip of the bill can reach 2.5 mm; and the primordium of the comb, the labial groove, and the uropygial gland are all well developed. We treated the embryos with GA on day 2.5 after fertilization, and each subgroup was observed on days 5.5, 9.5, and 21 (day-1 chick, HH Stage 46), respectively.

Briefly, the fertilized eggs were immediately transferred to an incubator (Haw-Yang Agricultural Farm, Taichung, Taiwan) and incubated at 37^o^C, RH 70%–80% for 1.5 days. To administer GA, the eggs were moved to a laminar flow chamber. A 2-mm^2^ hole was aseptically drilled through each egg shell, using a pin drill. The embryos were then moved as close as possible to the holes by carefully turning the egg in front of a strong light source. The subgroups were administered PBS (as the control), and 2, 6, 10, and 14 μM GA. The windows were then immediately sealed aseptically using 3M tape. The incubation was continued at 37^o^C and RH 70%–80%. Embryos were observed on days 5.5 and 9.5, and chicks were observed on day 1 (HH Stage 46) [22].

### Sample embryos

The chorioallantoic membranes, blood vessel, yolk, and egg white of fertilized eggs on day 5.5 and 9.5 were carefully removed. The embryos were successively rinsed several times with PBS and deionized water (DW). Photos were taken after the embryos were carefully dried using tissues.

### Sample Day-1 chicks

The day-1 (HH Stage 46) chicks were collected. The death rate and malformed rate were calculated. Blood was collected for counting. The alive day-1 chicks were euthanized under CO_2_-anethesia and the liver, pancreas, spleen, and necks of all chicks were excised, rinsed 3 times with PBS, dried using tissues, and weighed. Photos of the whole chicks and the organs were taken.

### Histochemical examinations

Organs were fixed in 10% formalin in PBS (pH 7.4) at 4°C for 24 h and processed for paraffin embedding. Paraffin sections were dewaxed in xylene and rehydrated in a series of ethanol washes. The nuclei of the specimens were subjected to Weigert’s hematoxylin eosin (HE) staining or the collagen content was stained with Sirius Red.

### Protein extraction from the cervical tissues

Frozen cervical tissues (approximately 100 mg) were homogenized using a homogenizer (T10 basic, The IKA Company, Germany) in 1 mL of Pro-PREP lysis buffer (pH 7.2). The homogenate was centrifuged at 12000 × g for 20 min at 4°C, and the supernatant was collected as tissue sample lysate. The protein content of lysate was determined according to the manufacturer’s instruction.

### Western blotting

The lysate-containing protein (50 μg) was heated at 100°C for 10 min before loading and separated on precast 7.5% SDS-PAGE. The proteins were electrotransferred onto a PVDF membrane in transfer buffer for 1 h. The nonspecific binding to the membrane was blocked for 1 h at room temperature with 5% nonfat milk in a TBS buffer. The membranes were then incubated for 16 h at 4^o^C with various primary antibodies. After extensive washing in the TBS buffer, the membranes were = incubated with secondary antibodies in blocking buffer containing 5% nonfat milk for 1 h at room temperature. The membranes were then washed with the TBS buffer and the signals were visualized using a Luminescent Image Analyzer LAS-4000 (Fujifilm, Tokyo, Japan). The gp130, leukemia inhibitory factor receptor (LIFR), GRB2, Ras, STAT, and PPAR-α were analyzed using immunoassay methods, according to the manufacturers’ instructions. β-actin was used as the reference protein.

### ELISA assay for IL-6, TNF-α, ROS, and MDA

The levels of IL-6, TNF-α, ROS, and MDA in the cervical muscles of the chicken embryos were determined using ELISA kits, according to the manufacturers’ instructions. A SYSMEX K-1000 Reader (San-Tong Instrument Co., Taipei, Taiwan) was used to read the optical density.

### Statistical analysis

Data obtained in the same group were analyzed using a Student’s *t* test and SPSS 10.0 statistical software (SPSS, Chicago, IL, USA). An analysis of variance and Tukey’s test were used to analyze the variance and identify significant differences between paired means. Significance determined by *p* < 0.05.

## Results and Discussion

### Effect of gallic acid in inducing malformation

**Fig 1:**
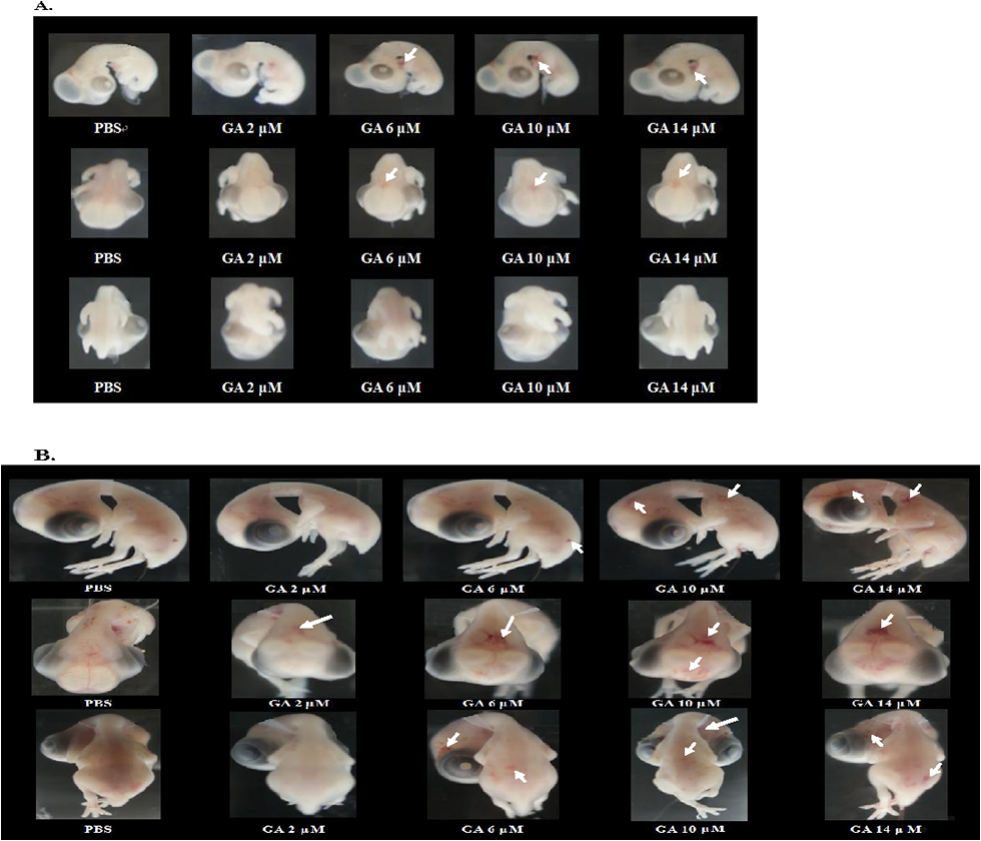
Dose-responsive gallic-acid-induced hemolysis in day-5.5 (A) and day-9.5 (B) chicken embryos. Cerebral hemolysis occurred early in day-5.5 (A) and more severely in day-9.5 embryos (B). Upper row: side view. Middle row: top view. Lower row: back view. Arrows indicate the hemorrhage sites (prevalence rate of hemorrhage: 5.5%–10.5% which is involved in the damaging rate of Table 1).

GA at 2 μM did not exert any damaging effect on the embryos (Fig 1A and 1B, Table 1). However, the still birth rates reached 16.6%, 44.1%, and 70.9% and the cervical muscle damage rates were 33.3%, 39.4%, and 29.2% at 6, 10 and 14 μM, respectively (Table 1). The prevalence of embryonic hemolysis ranged from 5.5% to 10.5% (involved in the malformation rate, as shown in Table 1), underlying the potential of GA teratogenicity to occur at doses ≥ 6 μM, corresponding to 1.02 mg/kg. The results revealed 10 μM GA to be the optimal dose, at which the peak damage rate of 39.4 ± 2.9% was observed (Table 1). By contrast, 14 μM GA caused the highest stillbirth rate of 70.9% and the damage rate decreased to 29.2% (Table 1). The cause of mortality might be strong prooxidant bioactivity. GA dose-responsively generates considerably more H_2_O_2_ in plain DMEM media than quercetin does [16, 23, 24], suggesting that GA is a stronger prooxidative antiproliferative than quercetin is regarding embryonic proliferation and differentiation.

**Table 1:** Damaging rate with pathological changes of musculus logissimus cervicis of day-1 chicks in dose-responsive manner.*

Group	Gallic acid (μM)	Normal rate (%)	Still birth rate (%)	Damaging rate (%)^*^
Control	PBS	100±0^a^	0±0^a^	0±0^a^
1	2	100±0^a^	0±0^a^	0±0^a^
2	6	50.2±11.2^b^	16.6± 0.0^b^	33.3±11.2^b^
3	10	16.6± 0.0^c^	44.1± 2.9^c^	39.4± 2.9^c^
4	14	0±0^d^	70.9±13.9^d^	29.2±13.9^d^

^*^Data calculated from day-1 chicks according to the occurrence of damaged musculus logissimus cervicis. The prevalence rate of holo-embryo hemolysis 5.5–10.5% (not shown) is involved in the damaging rate.

Data expressed in mean±SD (number of embryos n = 10 in each group).

The superscripts in lower case in each column indicate significantly dose responsive difference between groups.

### Intracerebral hemorrhage induced by gallic acid occurred early in day-5.5 embryos

GA induced teratogenesis at an early stage in the day-5.5 embryos (Fig 1A). Severe cerebral hemolysis was observed in day-9.5 embryos (Fig 1B). The occurrence was dose-responsive (Table 1, Fig 1A and 1B). Cerebral hemorrhage can be at least partially attributed to the strong prooxidant characteristic of GA [16, 23, 24]. An alternative cause is discussed in the following sections.

### The scoring of the pathological stages according to outer appearance

**Fig 2:**
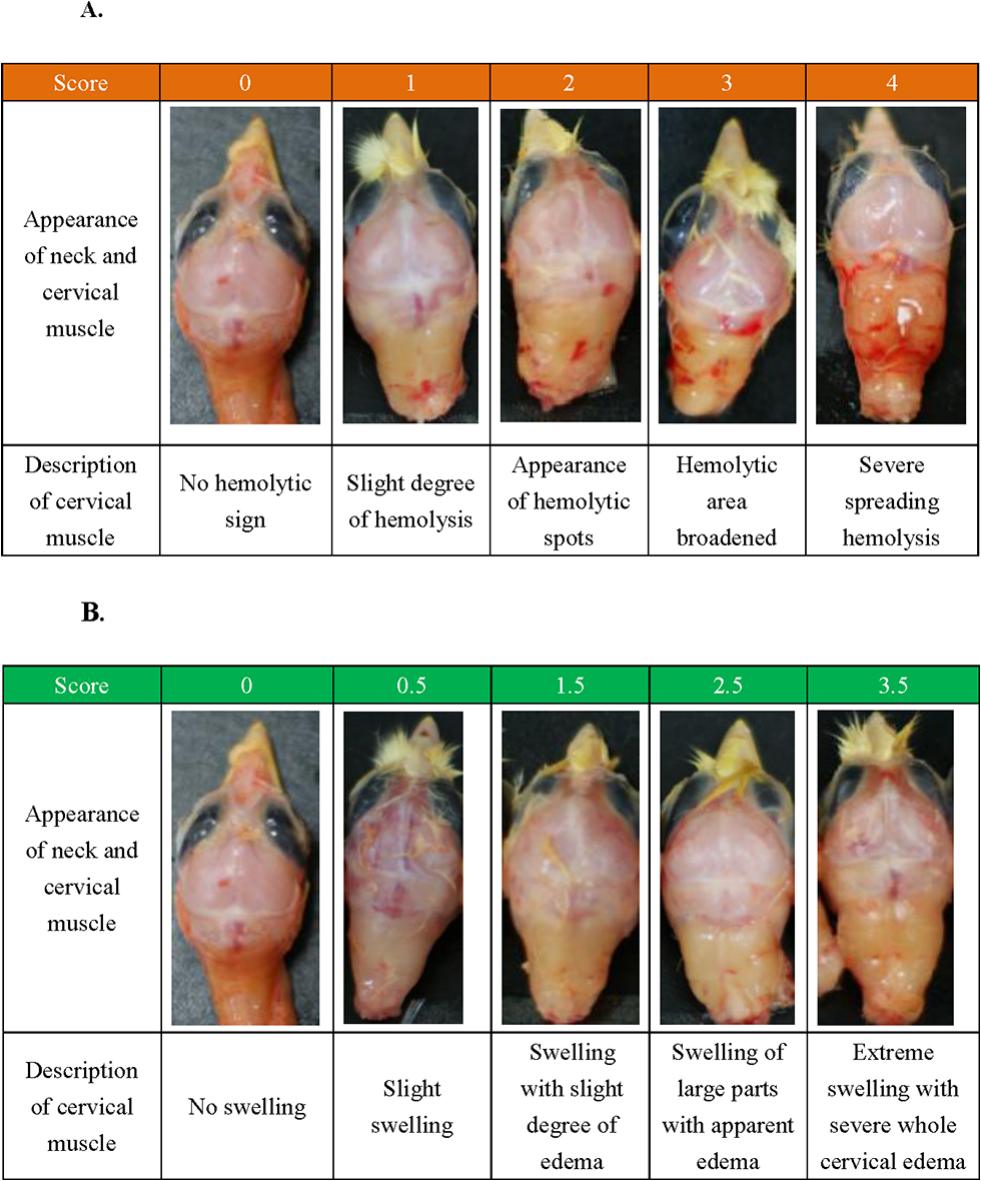
Scoring of the pathological changes of the musculus longissimus cervicis (the longest cervical muscle of the neck) in day-1 chicks. The extent of severity of edema and hemorrhage (A); and the scoring based on the degree of swelling and edema without hemorrhage (B).

The pathological scoring of the malformed changes was developed based on the degree of swelling, liposis (fat accumulation) of the musculi longissimus cervicis (the longest cervical muscle), and hemolysis in the cerebrum and cervical muscle (Fig 2A). However, as the actual prevalence rate of hemolysis in embryos reached only 5.5–10.5% which was included in the damaging rate (as addressed in the footnote of Table 1), we established a subscoring system based on the degree of edema, liposis, and hemolysis for further clssification of the status (Fig 2B).

### Body, liver, and spleen weights were affected by gallic acid

GA at low concentrations ≤ 2 μM behaved similarly to the control. At higher doses, GA retarded the development and growth of the embryos. The body weight of day-1 chicks was reduced in a dose-responsive manner from 44.7 g (PBS control) and 45.3 g (2 μM normal subjects) to 42.2, 35.3, and 32.4 g (*p* < 0.05) by GA at 6, 10, and 14 μM, respectively (Table 2).

**Table 2:** Fold changes of the oxidative stress related markes.*

Parameters	Gallic acid, μM				
	PBS control	2	6	10	14
IL-6, %	100	165±25^a^	210±46^b^	225±44^b^	234±38^b^
TNF-α, %	100	106±12	260±37^a^	285±33^a^	334±41^b^
ROS, %	100	102±13	187±27^a^	244±31^b^	305±42^c^
MDA, %	100	104±15	169±23^a^	228±35^b^	279±39^b^

*The data were calculated from triplicate experiment and expressed in mean±SD (n = 3).

Different superscripts in lower case indicate significantly different from each other in the same row.

In a previous study, F344 rats administered 10 μM GA exhibited significant loss not only in body weight [21] (Table 2), but also in liver weight in a dose-responsive manner, when compared with both PBS controls and subjects administered 2 μM GA. The liver weights decreased to 4.0, 3.8, and 3.2 g (*p* < 0.05) after GA treatment at 3, 5, and 7 μM, respectively (Table 2). In spleens, GA at 7 μM exhibited only an apparent weight-reducing effect (*p* < 0.05) (Table 2). Distinct organs exhibited varying susceptibilities to GA. Because the decreases in body and organ weights occurred simultaneously in a dose-responsive manner, the organ weight related to body weight did not differ in the various treatment groups (Table 2).

### Hematoxylin eosin staining exhibited moderate hemorrhaging and Zenker’s necrosis in the musculi longissimus cervicis

**Fig 3:**
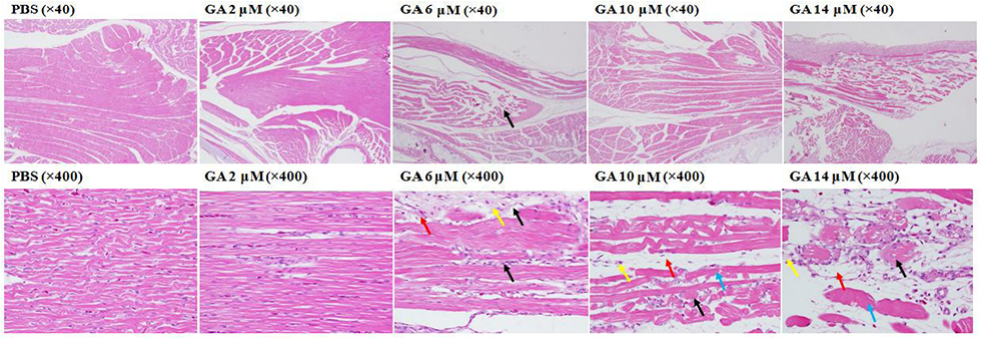
The HE stain of the musculus longissimus cervicis (cervical muscles) obtained from the normal and malformed embryos caused by various gallic acid concentrations. Magnification: ×40 (upper panel), ×400 (lower panel).

Typically, the organ most prominently damaged by GA was the cervix; therefore, HE staining was selectively conducted on the cervical muscles (musculus longissimus cervicis). GA at 10 μM caused only moderate hemorrhaging in the musculus longissimus cervicis. More severe pathological changes were observed in the 14 μM treated subjects, which exhibited severe hemorrhaging and Zenker’s necrosis in the cervical muscles (Fig 3). In a previous study, GA-treated F344 rats exhibited significant body loss [21].

### Blood and biochemical parameters affected by gallic acid

The white blood cell (WBC), eosinophil, lymphocyte, and monocyte counts significantly decreased after the uptake of GA (*p* < 0.05). The red blood cell (RBC) count was most severely affected by GA at 10 and 14 μM (*p* < 0.01). By contrast, the hemoglobin level was severely suppressed only at much higher doses of GA (e.g., 14 μM) (Table 3).

**Table 3:** The body- and organ-weight of day-1 chicks affected by different level of gallic acid.*

Parameters	Gallic acid, μM				
	Control	2	6	10	14
Body weight, g	44.7±3.8^a^	45.3±3.3^a^	42.2±3.4^b^	35.3±3.2^c^	32.4±3.6^d^
Liver, g	4.2±0.2^a^	4.2±0.2^a^	4.0±0.2^b^	3.8±0.3^c^	3.2±0.2^d^
Liver/BW,%w/w	9.4±1.3^a^	9.3±1.1^a^	9.5±1.1^a^	10.8±1.2^c^	9.9±1.1^b^
Spleen, g	0.1±0.0^a^	0.1±0.0^a^	0.1±0.0^a^	0.1±0.1^a^	0.07±0.0^b^
Spleen/BW, %w/w	0.24±0.01^a^	0.22±0.02^a^	0.24±0.01^a^	0.28±0.01^b^	0.22±0.01^a^

*Data expressed in mean±SD (n = 10 in each group). Different superscripts in lower case in each row indicate significant dose responsive difference between groups (*p*<0.05).

In a previous study, F344 rats administered GA (5 mM) exhibited a significant reduction of hemoglobin concentration, hematocrit level, and RBC count, and an increase of reticulocytes [21]. The suppressing effect of GA on RBC generation can be partially attributed to its prooxidant activity. The retarded transport and reduction of ferric ions can be the cause more relevantly to RBC production [25–27].

### *In vivo* characteristics of the erythrocytes affected by gallic acid

**Fig 4:**
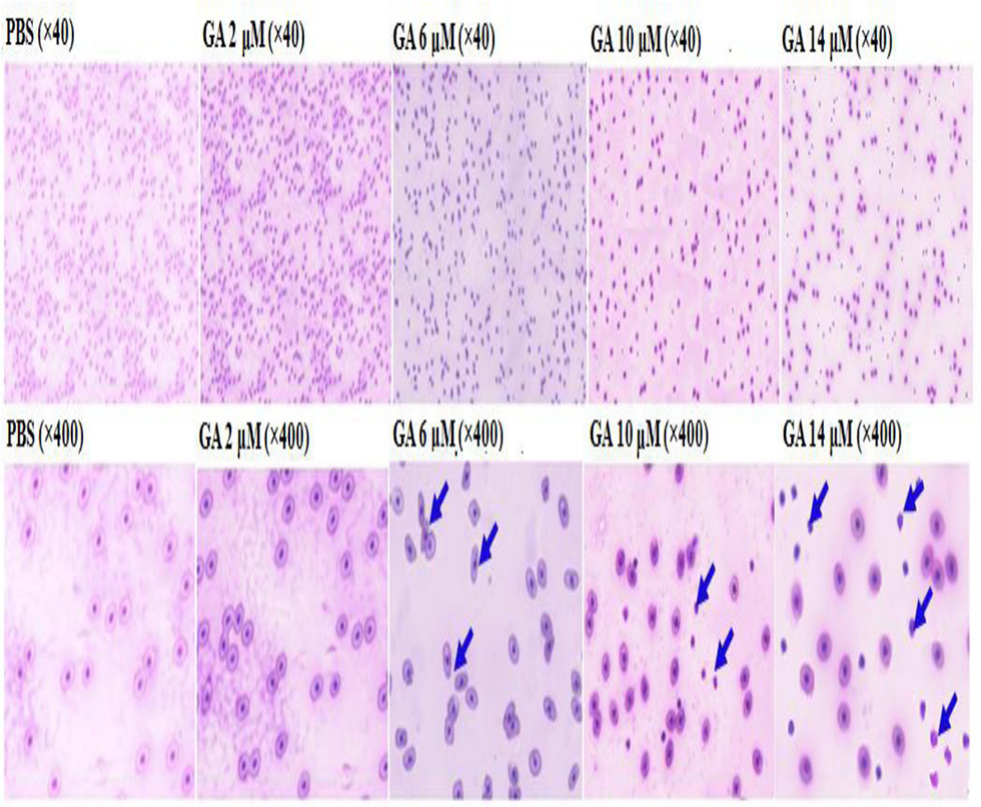
Light microscopic examination of *in vivo* RBC hemolysis induced by various gallic acid concentrations. The red blood cells were directly obtained from day-1 chicks treated with various gallic acid concentrations. As seen, the RBC’s that were elongated wgen ready to collapse (see pictures at 6 and 10 μM GA). The blue arrows indicate the nuclei released after collapsed.

The erythrocytes treated with 2 μM GA appeared to be similar in size and shape to those of PBS control subjects. Some RBCs in the GA (6 μM)-treated group were elongated or malformed, but did not exhibit collapse. GA at higher concentrations (e.g., 10 μM and 14 μM) induced severe RBC collapse and nuclear release (Fig 4). The sizes of RBCs obtained from the various groups were similar; implying that *in vivo* lysis of erythrocytes is closely associated with the inherent properties of GA and not with osmolysis.

### Gallic acid induced the generation of huge amounts of H_2_O_2_

GA dose-responsively generates considerably more H_2_O_2_ in plain DMEM media than quercetin does, and GA acts as a stronger prooxidant than quercetin does [16]. Autoxidation of GA increases radical intensity and changes the redox potential to a more oxidative state [28, 29], leading to tremendous formation of semiquinone and superoxide (•O_2_^**-**^) radicals [23, 24]. Hence, the hemolytic effect in embryos might be partially induced by the autooxidative behavior of GA, whereby large amounts of ROS can be generated [14]. Furthermore, the presence of Fe(II) or Fe(III) ions might accelerate the production of *in vivo* ROS through the coupled Haber-Weiss and Fenton reactions [30] S1 Text.

Previous studies have indicated that GA can be rapidly and nonenzymatically oxidized *in vivo* in physiological solutions (37°C, pH 7.4) to produce superoxide anions, H_2_O_2_ and GA quinones [31–33]. GA increases ROS levels, including •O_2_^-^, induces GSH depletion, and inhibits the growth of lung cancer and normal cells [34]. We showed that GA dose dependently enhanced the production of IL-6, TNF-α, ROS and MDA (Table 2). The level of IL-6 was the most apparently dominating starting from a dose as low as 2 μM, reaching 165±25, 210±46, 225±44 and 234±38% at GA 2, 6, 10, and 14 μM, respectivly comparing with the control (100%). Similar trend was found for TNF-α, ROS and MDA (Table 2). GA (but not quercetin) is also able to inhibit GJIC (a carcinogenic phenomenon) [16]. We suggest that at a blood pH value of 7.2 to 7.5, GA can be more rapidly oxidized in some oxygen-transporting organs, such as the lungs, erythrocytes, bone marrow, and spleen (Table 3). As mentioned, GA (5 mM)-treated F344 rats exhibited significant reduction of hemoglobin concentration, hematocrit levels, and RBC counts, and an increase of reticulocytes [21]. Extramedullary hematopoiesis, hemosiderin deposition, and congestion were histopathologically observed in the spleens, suggesting development of hemolytic anemia [21] (Table 4). In developing embryos, the smooth muscle membrane, the angiogenic system, and the blood cells actively develop and differentiate in numerous developing tissues, such as nerve tissue and internal organs and blood vessels, thereby indicating the target organs of GA cytotoxicity during embryonic development. In addition, GA failed to protect against H_2_O_2_-induced PC12 cell death, which can also be ascribed to the prooxidant potential and generation of ROS by GA [17, 23, 24]. GA at concentrations of up to 12 μM did not inhibit the peroxidation of phospholipids in the liposomal system [35]. Herein in our experiment, the levels of ROS and MDA were seen to increase repectively from 102±13 to 305±42% and from 104±15 to 279±39% (Table 2), implicating the failure of GA to protect the malformation like the hemorrhagic liposis of cervical muscles. Some antioxidants, such as ascorbic acid and *N*-acetylcysteine, were effective at protecting other cell lines [8], implicating distinct substrate-specific and cell-specific responses.

**Table 4:** Major blood and biochemical parameters affected by different level of gallic acid in day-1 chicks.*

Parameters	Gallic acid, (μM)				
	Control	2	6	10	14
WBC, 10^3^/μL	4.43±0.3^a^	4.44±0.3^a^	4.17±0.4^a^	4.13±0.5^a^	4.11±0.4^a^
RBC, 10^6^/μL	9.52±0.3^a^	9.50±0.2^a^	9.32±0.3^a^	9.10±0.3^b^	9.00±0.4^b^
Hb, g/dL	15.6±0.4^a^	15.4±0.3^a^	15.3±0.3^a^	14.4±0.4^b^	14.1±0.4^b^
Eosinolphils, %	1.0±0.5^a^	1.1±0.4^a^	1.5±0.6^d^	1.2±0.6^c^	0.7±0.7^b^
Lymphocytes, %	81.1±2.8^a^	82.1±2.5^a^	74.8±3.3^b^	74.4±3.5^b^	74.3±3.6^b^
Monocytes, %	1.0±0.5^a^	1.1±04^a^	1.7±0.4^b^	2.1±0.3^c^	2.2±0.3^c^

*WBC: white blood cells. RBC: red blood cells. Hb: hemoglobin. Data expressed in mean±SD from triplicate experiments. (n = 10). Different superscripts in lower case in each row indicate significantly different between each other (*p*<0.05).

### PPAR-α was suppressed by gallic acid in a dose responsive manner

**Fig 5:**
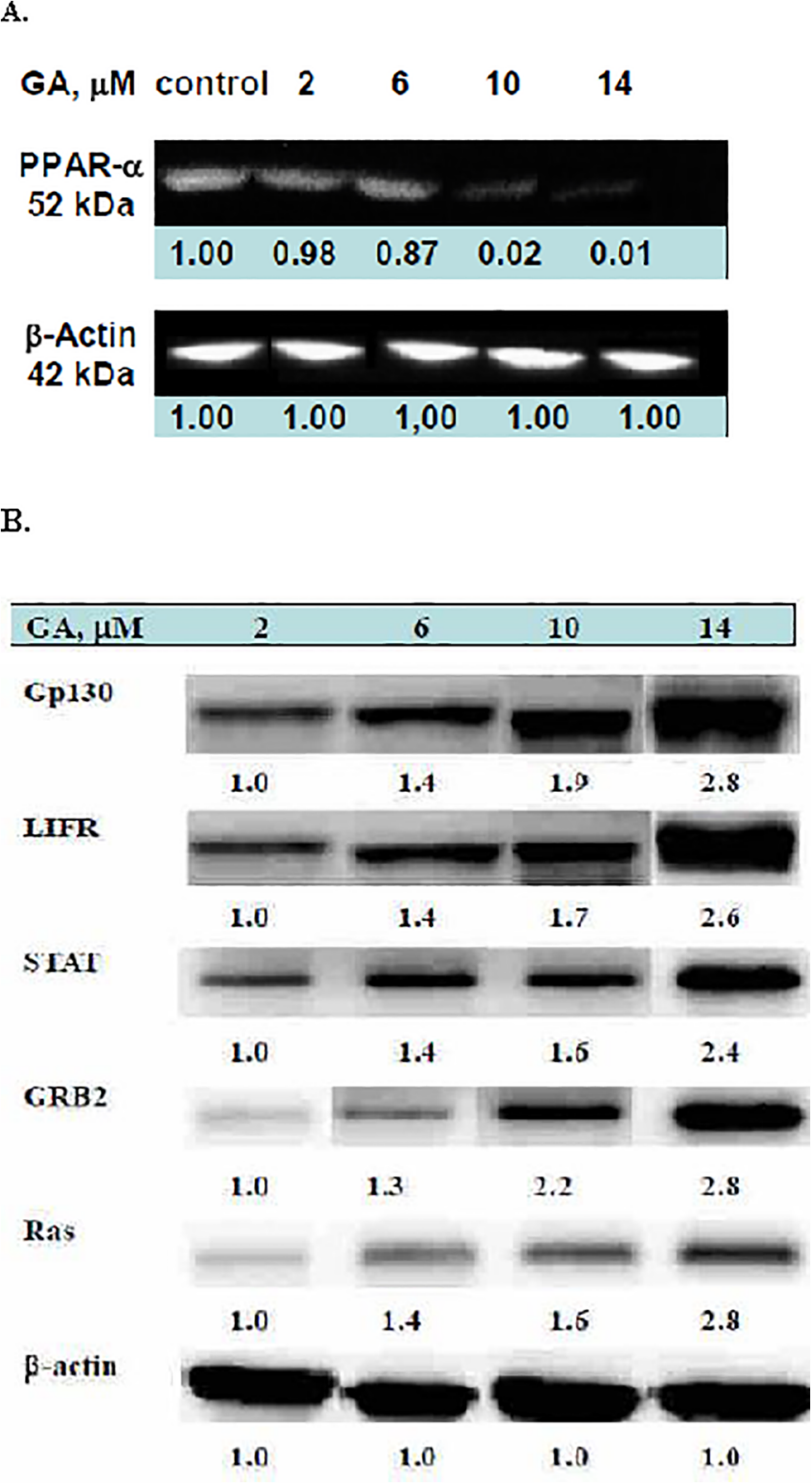
(A) Western blotting of the PPAR-α expression. The doses of GA used were 2, 6, 10, and 14 μM. (B) The Ras/Raf-mediated growth inhibition and differentiation induced by gallic acid. Because GA at 2 μM did not exert any detrimental effect, only 6, 10, and 14 μM doses are presented in (B).

The changes in PPAR-α explained why liposis accompanied hemolysis in the musculi longissimus cervicis when the chicken embryos were treated with GA; GA downregulated the level of PPAR-α in the musculi longissimus cervicis in a dose-responsive manner. The suppression ratios reached 1.00 (control), 0.98 (2 μM), 0.87 (6 μM), 0.02 (10 μM), and 0.02 (14 μM) (Fig 5A), indicating that the β-oxidation of fatty acid was also retarded in a dose-responsive manner; hence, a large amount of fat was accumulated in the musculi longissimus cervicis (Fig 2B). PPARα is expressed in liver, kidney, heart, muscle adipose tissue, and others [36]. PPARα is able to improve insulin sensitivity [37]. As cited, all nutraceutic groups except quercetin were characterized with suppressed PPARα [38]. Insulin resistance is often associated with increased levels of intracellular triglycerides, diacylglycerol and decreased fat β-oxidation [38]. Conversely, quercetin retains full strength PPARα and is effective in acting as an antihypercholesterolemic, indicating that the complex hypercholesterolemia of CKD is controlled not only by mitochondrial fat oxidation [39]. The parallel modulation pattern of PPARα and insulin implicates a close link between these 2 parameters [39].

### Gallic acid inhibited growth and differentiation by activating the gp-130/LIFR/GRB2/Ras/JAK/STAT pathway

**Fig 6:**
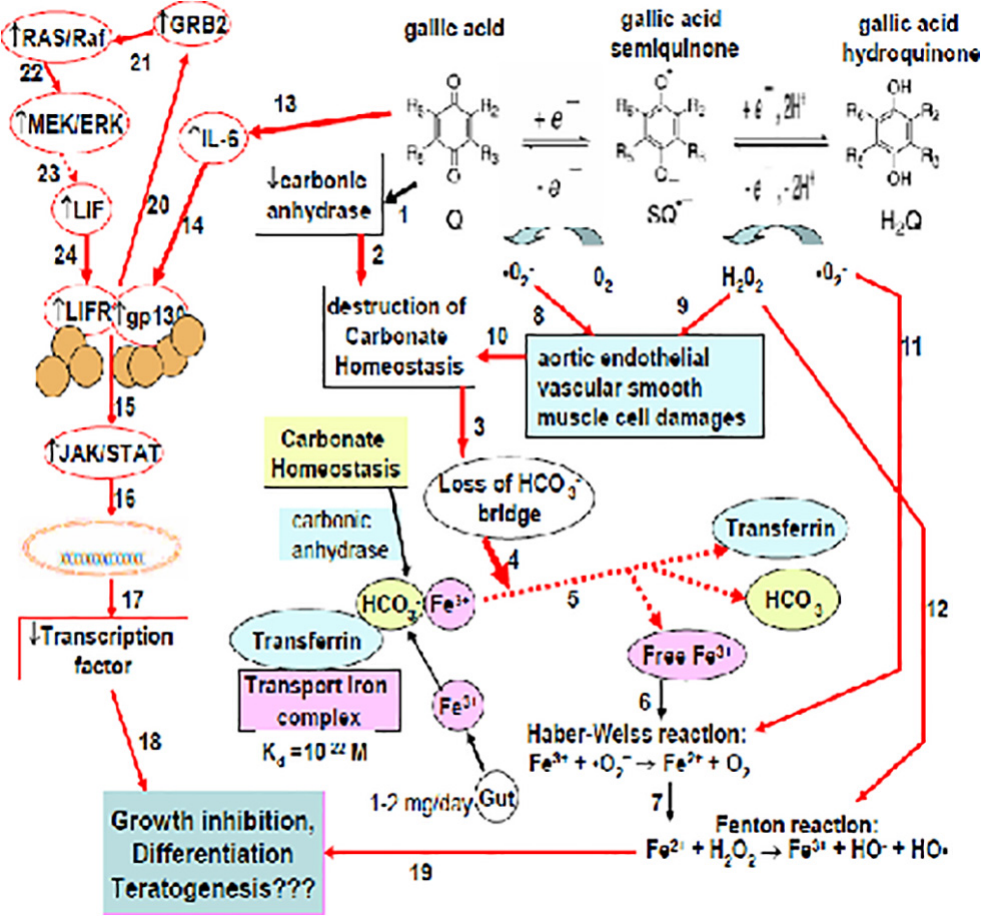
Proposed teratogenicity of gallic acid. GA directly inhibits carbonic anhydrase, resulting in the destruction of carbonate homeostasis (Reactions I and II). The transport of ferric ions (Fe^3+^) by transferrin requires a carbonate bridge (dissociation constant K_d_ = 10^–22^ M [25]) for the whole complex formation; therefore, the destruction of carbonate homeostasis (Reaction III) leads to negligible ferric ion transport (Reaction V). GA is a stronger prooxidant than quercetin [16]. The autooxidation of GA produces large amounts of superoxide anions, hydroxyl free radicals, and hydrogen peroxide, which alternatively injures aortic smooth muscle cells (Reactions VIII and IX), which, in turn, accelerates more readily the destruction of carbonate homeostasis (Reaction X) and triggers (Reaction IV) the decomposition of transferrin-bicarbonate-iron complexes (Reaction V). The released free ferric ion reacts with the superoxide anion (the Harber-Weiss reaction; Reaction XI) [14, 45], producing large amounts of ferrous ions (Fe^2+^), which rapidly undergo the Fenton reaction in the presence of hydrogen peroxide to yield additional hydroxyl free radicals [17] (Reaction XII).

The western blot analysis revealed that the signal proteins gp130, leukemia inhibitory factor receptor (LIFR), growth factor receptor-bound protein 2 (GRB2), Ras, and STAT were all upregulated by GA in a dose-responsive manner (Fig 5B), implicating the signal transduction pathways of Ras/Raf-mediated-growth inhibition and differentiation. Ras protein regulates diverse cell behaviors and is involved in the MAPK/extracellular signal-regulated kinase (ERK) pathway of signal transduction [20]. We suggest that the activation of Ras/Raf could be the alternative GA toxicity, and our data (Fig 5) were consistent with those of Lo et al. [20] and Liu et al. [40]. Sustained activation of the Ras/Raf/MEK/ERK pathway can lead to cell cycle arrest in numerous cell lines [41]. However, the proliferative marker PCNA has been suggested to be directly related with the proliferation [42], futher work will be conducted to confirm this aspect. A summary of the signal pathway is shown in Fig 6. Previous studies have indicated that the biological actions of IL-6, leukemia inhibitory factor (LIF), and ciliary neurotrophic factor (CNTF) are mediated by respective functional receptor complexes (FRCs) consisting of a common signal-transducing component, gp130. The LIF is a polyfunctional cytokine that affects the differentiation, survival, and proliferation of a wide variety of cells in adults and embryos [43, 44]. Both LIFR and gp130 include components of the receptors for the majority of hematopoietic cytokines [45], further implicating the alternate role of GA in inducing embryonic hemolysis (Fig 1). Furthermore, the upregulation of receptor subunits in FRCs in distinct cell populations plays a critical role in the effective regeneration of both myofibers and motor neurons [19], underlying the teratogenicity exerted by GA in the embryonic cervical muscles. Moreover, the inhibition of carbonic anhydrase might cause the dissociation of the transferrin-carbonate-ferric ion complex, causing a severe hemolytic response [26, 27]. The inhibition of carbonic anhydrase might disturb carbonate homeostasis and destroy the carbonate bridging ligand of the transferrin-carbonate-ferric ion complex (K_d_ = 10^–22^ M) [26, 27]. Without the anion cofactor, iron binding to transferrin is negligible [25] (Fig 6). Hence, a large portion of ferric ions might remain in a free ion state, which, in turn, can accelerate the coupling of Harber-Weiss [46] and Fenton reactions and enhance the cyclic hydroxyl free radical oxidative stress on cell proliferation [17] (Fig 6; see the Supplement).

Polyphenols are broadly considered beneficial to human health because they can scavenge free radicals and ROS [47]. However, some polyphenols can simultaneously act as both scavengers and ROS forming agents [48]. Heavily reducing species such as pyrogallol (3 adjacent phenol groups) containing (–)-epigallocatechin (EGC) and (–)-epigallocatechingallate (EGCG), typically produces superoxide radicals from molecular oxygen [49]. Orthoquinones formed by the loss of 2 electrons from pyrogallol and catechol (2 adjacent phenol groups) moieties can biochemically participate in enzymatic redox cycling reactions, which can produce superoxides and other ROS [50]. The majority of these redox reactions can exert a determinant influence over many biological functions, such as cell cycle and apoptosis, in which the pyrogallol group typically plays a crucial role [51].

The potential growth-inhibitory signaling by Ras/Raf was activated. The autocrine-paracrine signaling diverges from the intracellular signaling distal to MEK/ERK. The extracellular pathway mediates its effect through LIF expression and activation of JAK-STAT3.

The apoptosis induced by GA is atypical [14]. Although VSMCs are positive for *in situ* nick end-labeling (TUNEL), they do not exhibit a DNA ladder pattern in gel electrophoresis and are negative for *Taq* polymerase-based *in situ* ligation, which is more specific for apoptosis than for TUNEL. Moreover, GA-induced cell death is not prevented by Boc-Asp-fmk (a pan-caspase inhibitor) [14], indicating that neither the intrinsic nor extrinsic mitochondrial pathways are involved in GA-induced teratogenicity and hemolysis. Additionally, the treatment of PC12 cells with GA results in the upregulation of phospho-JNK expression [17].

In summary, a dose of GA > 0.34 mg/kg potentially exhibits teratogenicity. The teratogenic rates reach 33.3%, 39.4%, and 29.2% at GA doses of 6, 10, and 14 μM, respectively, involving 5.5%–10.5% of holohemolysis (not specifically shown, but involved in the malformation rate). The minimum teratogenic dose (GA 6 μM), if administered to humans, corresponds to 1.02 mg/kg of body weight. In examining the malformation and teratogenicity, GA at 10 μM was observed to be the optimal dose, which elicited the maximum malformation rate and the lowest still birth rate. The targeted tissue was typically that of the musculi longissimus cervicis (the longest cervical muscle). Zenker’s necrosis, liposis, and severe edema with focal infiltration were frequently observed and the body weight decreased severely. The organs or organelles primarily affected were the liver, spleen, erythrocytes, hemoglobin, eosinophils, lymphocytes, and monocytes (Table 4). The Ras pathway was significantly upregulated, indicating that the transcription signaling pathway was severely affected. We suggest that the likely causal effect of GA in inducing teratogenic births might be any of the 3 main pathways, or by synergism: i) The upregulation of the Ras/Raf/JAK/STAT pathway triggered the transcriptional signals to inhibit embryo growth and differentiation; ii) The direct attack of prooxidative hydroxyl free radicals on the proliferating endothelial smooth muscle in many developing organs [14]; and iii) Carbonic anhydrase was inhibited by GA, destroying the carbonate homeostasis [26, 27]. The carbonate bridging ligand of the transferrin-carbonate-ferric ion complex has a dissociation coefficient K_d_ = 10^–22^ M. When the dissociation coefficient is destroyed, the released ferric ions accelerate the Harber-Weiss reaction [46] to be coupled by the Fenton reaction, cyclically producing large amounts of hydroxyl free radicals, exerting substantial oxidative stress on the proliferating and differentiating cells [17]. The results are summarized in Fig 6. Thus, although it is generally believed that antioxidants are beneficial to health, individual antioxidants might exert either positive or negative effects, depending not only on their doses and antioxidative bioactivities, but also on the extent of their prooxidative characteristic.

## Conclusion

GA at doses ≥ 6 μM (≥ 1.02 mg/kg) potentially exhibit teratogenicity and cause cerebral hemorrhage and cervical muscle liposis. This amount of GA can be easily consumed by a pregnant woman in teas, causing a risk to the fetus. The tissues and cells most affected are the cervical muscles and erythrocytes. The mechanism of action of GA is multidisciplinary: Liposis can be ascribed to the downregulation of PPAR-α; erythrocyte hemolysis can be attributed to the unique autooxidative prooxidant behavior of GA and the inhibition of carbonic anhydrase; and proliferation and differentiation deficits can be attributed to the upregulation of the Ras/Raf/JAK/STAT pathway.

## Supporting Information

S1 Text Autooxidation of gallic acid and *in vivo* coupling of the Haber-Weiss and Fenton reactions. Gallic acid induced the generation of huge amounts of H_2_O_2_, the presence of Fe(II) or Fe(III) ions might accelerate the production of *in vivo* ROS through the coupled Haber-Weiss and Fenton reactions.
(DOC)
