# Supplementary material for: The Teratogenicity and the Action Mechanism of Gallic Acid Relating with Brain and Cervical Muscles
Source: PLoS One. 2015 Jun 1;10(6):e0119516. doi: 10.1371/journal.pone.0119516 (PMC4452303; doi:10.1371/journal.pone.0119516)
Supplement: S1 Text — Gallic acid induced the generation of huge amounts of H2O2, the presence of Fe(II) or Fe(III) ions might accelerate the production of in vivo ROS through the coupled Haber-Weiss and Fenton reactions. (DOC) [file pone.0119516.s001.doc]

**S1_Text.doc**

GA has been reported to increase radical intensity and change the redox potential to a more oxidative state [28, 29]. The detailed mechanism responsible for this effect of GA has not been elucidated. Polyphenolics can exert prooxidant effects through autoxidation. The initial step leads to semiquinone and superoxide (•O_2_**^-^**) radical formation [23, 24]. The adverse hemolysis effect (on embryos) might be due to the autooxidation of GA: the ROS, including hydrogen peroxide (H_2_O_2_) and superoxide anion (•O_2_^-^) [14]. Alternatively, the pH value might alter the chemical activity of GA, which is more pronounced in the pH range of 6 to 12, whereas the presence of Fe(II) or Fe(III) exerts a slight prooxidative effect [30]. In the presence of Fe(II) or Fe(III) ions, *in vivo* ROS are produced by 2 coupled reactions: the Haber-Weiss reaction (Eq. I),

Fe^3+^ + •O_2_^−^ → Fe^2+^ + O_2_………………...........................…(I)

and the Fenton reaction (Eq. II),

Fe^2+^ + H_2_O_2_ → Fe^3+^ + HO^-^ + HO•.......................................(II)

and simultaneously through cyclic autoxidations:

H_2_O_2_ + •OH → H_2_O + •O_2_^−^+ H^+^ .........................................(III)

H_2_O_2_ + •O_2_^−^→ O_2_ + OH^−^ + •OH…………………………..(IV)

These reactions were observed *in vivo* and are a possible source of oxidative stress. Nonetheless, Reactions III and IV exhibit negligible rate constants. Moreover, GA dose-dependently generates considerably more H_2_O_2_ in DMEM media without cells than quercetin does. GA exhibited stronger antiproliferative activity than quercetin did in both Caco-2 human colon cancer cells (Caco-2 cells) and WB-F344 normal rat liver epithelial cells (WB cells) [16].

Previous studies have indicated that GA is rapidly and nonenzymatically oxidized in physiological solutions (37 °C, pH 7.4), generating superoxide anions, H_2_O_2_ and GA quinones [32]. Superoxide anions (by destroying NO) and low H_2_O_2_ levels (by activating cyclooxygenase) both increased the vascular tone of intact and rubbed aortic rings in WKY rats; moderate H_2_O_2_ levels decreased vascular tone; and high H_2_O_2_ and GA quinone levels caused irreversible relaxation because of cellular damage [32]. Similar results (at > pH 7) were reported by Friedman and Jurgens [31]. Blood pH values range from 7.2 to 7.5, suggesting that GA can be oxidized more rapidly in oxygen-transporting organs, such as the lungs, erythrocytes, bone marrow, and spleen.

In a cell-free system, GA produces •OH in the presence of Fe^2+^-EDTA, which is quenched by CAT and DFX, suggesting involvement of the Haber-Weiss reaction [14]. Moreover, GA-induced death in several cell lines can be inhibited by antioxidants such as ascorbic acid, *N*-acetyl-l-cysteine, catalase, and superoxide dismutase (SOD) [8]. Thus, the prooxidant potential of GA has recently been suggested in addition to its antioxidant property.

Conversely, the failure of GA to protect against H_2_O_2_-induced PC12 cell death is attributed to the generation of ROS by GA [17]. GA-induced lung cancer cell death might be caused by GSH depletion as well as ROS level changes [4]. As mentioned, polyphenolics can exert prooxidant effects through autoxidation, in which the initial step leads to semiquinone and superoxide (•O_2_**^-^**) radical formation [23, 24]. These radicals are highly reactive and can damage major macromolecules, such as proteins or DNA. Moreover, both radicals accelerate autoxidation, generating H_2_O_2_ [52–54]. The presence of transition metals or H_2_O_2_ can reduce the antioxidant capacity of phenolics.

GA (but not quercetin) inhibits GJIC (a carcinogenic phenomenon). All the aforementioned adverse effects can be partially prevented by the addition of catalase [16]. In developing embryos, the proliferation of smooth muscle membranes differentiates intensively in numerous developing tissues, such as nervous tissue, internal organs, and blood vessels, indicating the targets of GA cytotoxicity in developing embryos.
